# Searching for epilepsy’s crystal ball

**DOI:** 10.7554/eLife.29853

**Published:** 2017-07-26

**Authors:** Olli Gröhn, Alejandra Sierra

**Affiliations:** A.I. Virtanen Institute for Molecular Sciences, University of Eastern Finland, Kuopio, Finlandolli.grohn@uef.fi; A.I. Virtanen Institute for Molecular Sciences, University of Eastern Finland, Kuopio, Finland

**Keywords:** MRI, biomarker, brain injury, epilepsy, inflammation, plasticity, Human, Mouse

## Abstract

Multi-modal MRI techniques have identified biomarkers that could help to predict whether someone will develop epilepsy.

**Related research article** Janz P, Schwaderlapp N, Heining K, Häussler U, Korvink JG, von Elverfeldt D, Hennig J, Egert U, LeVan P, Haas CA. 2017. Early tissue damage and microstructural reorganization predict disease severity in experimental epilepsy. *eLife* **6**:e25742. doi: 10.7554/eLife.25742

Will you have epilepsy in ten years as a result of hitting your head in a car accident last week? Currently, no one can answer this question because there is no known biological characteristic that we can measure to determine whether someone will develop epilepsy or not. The discovery of such a biomarker could accelerate the development of drugs and personalized treatment plans for patients with epilepsy.

More than 50 million people have epilepsy, which often develops slowly after a trigger such as a stroke, brain tumor or traumatic brain injury. Now, in eLife, Pierre LeVan, Carola Haas and colleagues at the University of Freiburg and the University of Karlsruhe – including Philipp Janz, Niels Schwaderlapp and Katharina Heining as joint first authors – report that they have used a combination of magnetic resonance imaging (MRI) techniques to look inside a brain that is in the process of developing epilepsy (Janz et al., 2017). This has revealed a number of structural and metabolic changes that could act as predictive biomarkers.

While MRI has been widely used in clinics and hospitals for decades, researchers still constantly develop new MRI techniques (Lerch et al., 2017). Advanced diffusion MRI, for example, uses variable strong magnetic fields to track the diffusion of water molecules through the body’s tissues. This diffusion is hindered by many biological boundaries, such as cell membranes, and so can provide information about the microstructure of tissues and organs (Figure 1A). The Human Connectome Project, for example, has used advanced diffusion MRI to map the large bundles, or tracts, of nerve fibers that connect different brain regions. However, advanced diffusion MRI can also be used to evaluate subtle changes at the microstructural level that affect populations of cells (Hutchinson et al., 2017).

**Figure 1.**
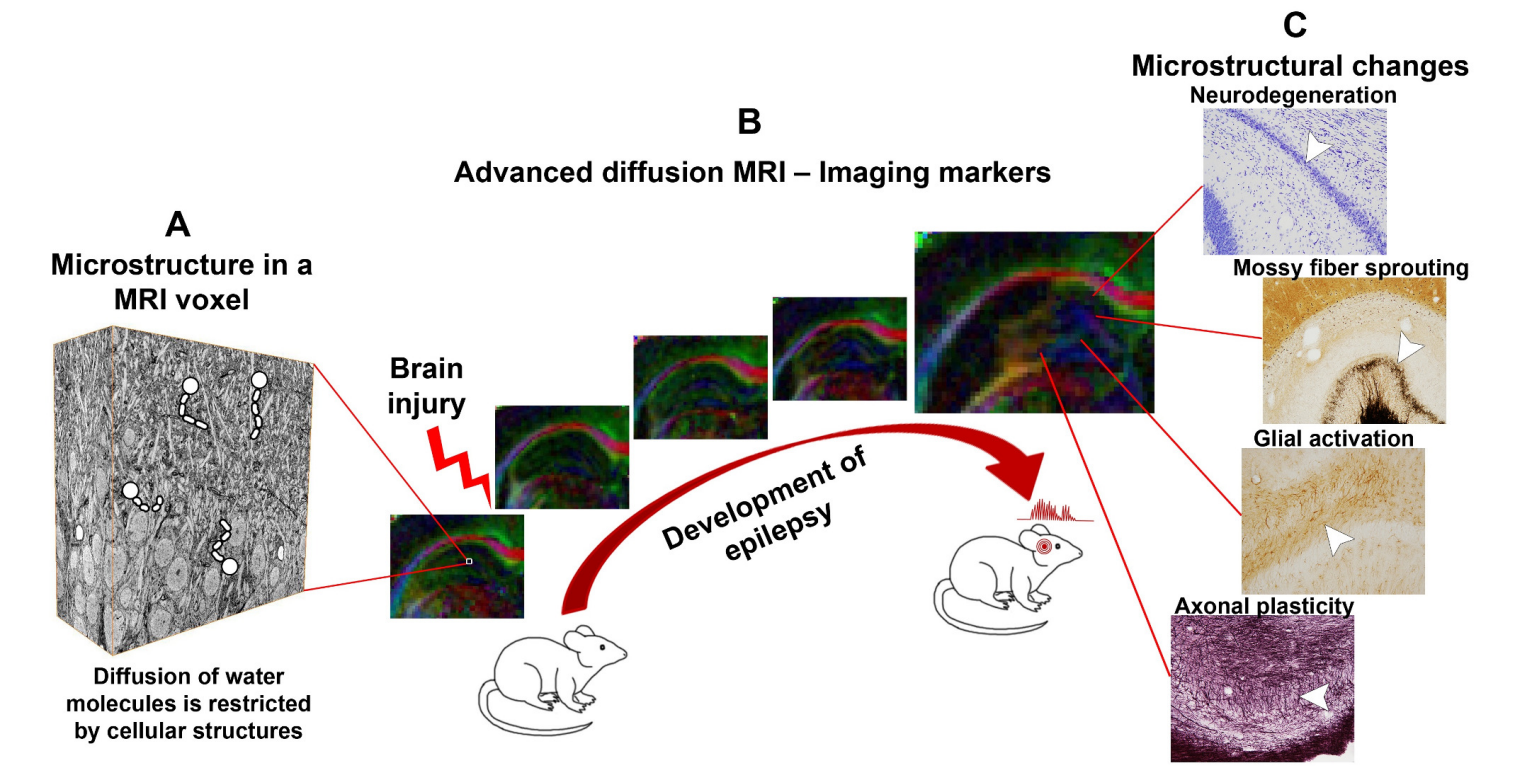
Advanced diffusion MRI can reveal changes in the brain that occur as an individual develops epilepsy. (**A**) Complex tissue microstructure in an MRI voxel of the rat hippocampus, as visualized by 3D electron microscopy. Cellular structures in the tissue hinder the diffusion of water molecules. (**B**) Advanced diffusion MRI can probe the microstructural information contained within a voxel by detecting the diffusion of water molecules. As epilepsy develops after a brain injury (left to right), the properties of the brain tissue in the hippocampus change, altering the diffusion of the water molecules. The colors in the maps represent the orientation of diffusion (blue represents up-down orientation, red is left-right, and green front-back). (**C**) The diffusion MRI data can be used to visualize cellular level changes – including cell death, mossy fiber sprouting, the activation of glial cells, and axonal plasticity – that occur as epilepsy develops after different brain injuries. These changes can be visualized by staining the tissue and studying it under a microscope. In the future, this information could help to predict whether epilepsy will develop after a brain injury.

MRI images are made of a series of 3D pixels (called voxels), each of which represents an average measurement calculated over a volume of 110×110×500 µm^3^ (in the example in Figure 1B). We are just starting to understand how the information that diffusion MRI reveals about the microstructure of the brain relates to the information about cell types and cellular components that is revealed by other techniques such as 3D electron microscopy (Figure 1A). Nevertheless, we already know that diffusion MRI can detect cellular level changes in a part of the brain called the hippocampal formation in rats that display the hallmarks of temporal lobe epilepsy (Kuo et al., 2008; Laitinen et al., 2010; Salo et al., 2017; Figure 1C).

Janz et al. build on this foundation by studying the brain of a mouse model of human temporal lobe epilepsy. They demonstrate that by combining information from advanced diffusion MRI and more conventional MRI techniques – quantitative T2 MRI and magnetic resonance spectroscopy – it is possible to assess damage in the hippocampus as early as the first day after an epilepsy-inducing injury. Importantly, these changes correlated with longer-term cell death and microstructural changes in the hippocampus, which are hallmarks of temporal lobe epilepsy, and can potentially be used to predict the severity of epilepsy and the appearance of seizures.

In a particular highlight, Janz et al. evaluated in detail the microstructural changes that occur in the hippocampal formation as epilepsy develops. To perform this, they collected data using a variant of an advanced diffusion MRI methodology called HARDI (short for high angular resolution diffusion imaging), and then exploited processing methods to reconstruct the data. The imaging data was detailed enough to show that different microstructural changes occur in different hippocampal subfields as epilepsy develops. The extent of these subfield-specific changes correlates with how severely the individual will be affected by epilepsy. More importantly, Janz et al. could associate these changes with specific cell types, such as the diffusion of water being restricted by the reorganization of glial cells.

These results are extremely valuable for clinical use because they show that microstructural changes in the brain can be used to predict how epilepsy will develop, and also reveal the cellular processes that caused these changes. Indeed, Janz et al. found similar microstructural changes in human hippocampal tissue specimens obtained from patients who had undergone surgical treatment for temporal lobe epilepsy.

Epilepsy can develop in a wide variety of ways in humans. To reflect this variation, the results presented by Janz et al. will need to be validated in larger groups in different animal models, and then finally tested in human patients. New biomarker candidates for epilepsy are in high demand (Pitkänen et al., 2016), but it is unlikely that structural MRI can present a candidate that is sensitive and specific enough to work on its own. However, the work of Janz et al. could form one part of a combinatory approach. One day, such approaches could help us to predict whether someone will develop epilepsy (and how severely), and guide their treatment.
